# Role of social media tools in online teaching: perception of physiotherapy students and knowledge translation

**DOI:** 10.1186/s43161-021-00065-5

**Published:** 2022-02-23

**Authors:** G. Shankar Ganesh, Mrutyunjaya Mishra, Narendra N. Dalei, Shabana Khan, Rajeev Ranjan, Sapna Dhiman

**Affiliations:** 1 Composite Regional Centre for Skill Development, Rehabilitation, and Empowerment of Persons with Disabilities, Lucknow, Uttar Pradesh 226017 India; 2 Department of Hearing Impairment, Dr. Shakuntala Misra National Rehabilitation University, Lucknow, Uttar Pradesh 226017 India; 3 Department Economics and International Business, School of Business, UPES, Dehradun, India; 4 Sign Language Interpreter, La Martiniere College, Lucknow, 226001 India; 5 Delhi Pharmaceutical Sciences and Research University, New Delhi, 110017 India

**Keywords:** E-learning, Knowledge translation, Social media

## Abstract

**Background:**

During the COVID-19 lockdown period many education institutions have shifted their focus from the traditional face-to-face education to online instruction mainly through various social media (SM) tools. However, it is not known if these results can be generalized across locations where infrastructure facilities are unevenly distributed. Further, no previous work has explored the role played by SM tools in knowledge translation. The objectives of this work are 1. To evaluate the students perceptions on the accessibility and acceptability of SM tools via an anonymous online survey and 2. Assess the efficacy of SM tools as an educational medium in imparting knowledge change.

An online survey using an anonymous web-based questionnaire was conducted to assess the student’s accessibility and acceptability of SM tools as a direct information sharing pathway between the faculty and students. A randomized comparative design was utilized to evaluate knowledge change via an online examination administered 10 min before and after an online class delivered via 2 different SM platforms (Google meet, YouTube) and e-mail.

**Results:**

Data were obtained from 627 participants through a survey. Though 71.1% of the respondents believed online classes have helped them in their study, only 21.4% and 22.6% of the participants strongly agreed that social networking platforms are helpful for teaching and will be used for teaching/learning in the future respectively. The ANOVA responses to evaluate knowledge transfer from 224 participants who were randomized to receive course content through Google meet, YouTube, and e-mail showed no significant differences in outcomes before and after the delivery of contents.

**Conclusion:**

Our findings suggest that multiple external and internal factors need to be addressed before substituting classroom teaching with online teaching, especially during emergencies.

## Background

The web has grown from a hyperlinked collection of read-only information to the inclusion of resources that can facilitate online discussion, participation, and sharing of various forms of content. Such media seems optimal to transform the method of teaching and learning. Social media (SM) is defined as a ‘collection of web-based technologies that share a user-focused approach to design and functionality, where users can actively participate in content creation and editing through open collaboration between members of communities of practice’ [1]. SM can reach a wider demographic of recipients compared with other conventional methods such as radio broadcasting, print media (i.e. newspapers and journals), and books [2]. Various SM tools such as those that involve social networking (such as Facebook and Twitter), video platforms (such as YouTube and Facebook), and blogging and microblogging sites are available. The increase in accessibility to the internet and mobile technologies has made SM tools a mainstream means for information sharing.

SM tools are increasingly being used in the educational sector. Virtual connections and open communication offered by SM tools provide an ideal and collaborative learning environment for students [3]. SM tools such as Facebook and YouTube have been reported to increase students’ knowledge and learning outcomes [4]. SM tools promote informal learning by providing more than one channel to communicate with other parties for accessing course content and video clips, sharing instructions and notes, and student engagement [5, 6]. The introduction of SM in academics has led to the development of new pedagogical practices such as student publication, social learning, and dynamic learning resources [7]. The educational benefits of these tools are well documented in literature [8, 9]. Other documented benefits of SM in education include positive learning experiences [1, 10] and an increase in knowledge and skills [11]. Despite the beneficial role of SM tools in education, scientific literature highlighting the impact of SM has been criticised for lack of methodological rigour [1], and it remains focused on culture change and different aspects of the learning experience [1, 12]. Further, whether SM enables deeper learning needs to be explored.

During COVID-19 pandemic, which was declared by the World Health Organisation as a public health emergency, the Government of India implemented a countrywide lockdown from March 2020, and it was extended in five phases. During the lockdown period, schools and colleges were closed, and thus, several of these institutions moved from traditional face-to-face education to online instruction. This transition has been challenging for teachers as well as students because it necessitates embracing the online teaching-learning process [13]. The online medium of instruction means that faculty members teach and students learn through electronic devices connected to the internet from their respective residences.

Several studies have examined the effect of online teaching and learning during this pandemic; the medical education community in India seems to have endorsed online teaching. Shetty et al. [14] evaluated the attitude of medical undergraduate students towards online learning in the subject of ENT through the student portal of their university website during the pandemic. The results showed that online learning was favoured by students during the pandemic, despite the presence of several technical and other barriers. Similar views favouring medical online education have been expressed by members of the medical community within as well as outside India [15, 16]. Mahdy [17] evaluated the effect of COVID-19 pandemic on academic performance and online learning during lockdown on veterinary medical students and researchers. The author concluded that the academic performances of participants were affected during the pandemic, and it is difficult to fulfil veterinary competencies as most of the subjects are practical.

Data collected from undergraduate physiotherapy students in a university in Saudi Arabia showed that both male and female students had positive attitudes towards using SM platforms for learning [18]; however, it is not yet known if students belonging to physiotherapy discipline in India are prepared to accept this forced change, as it may be difficult for students to ‘unfreeze’ the traditional teaching-learning [19] for using SM tools for learning.

In a scoping review [20], the authors recommended that more studies should explore students’ perspectives and attitudes towards the use of SM in learning and teaching. Therefore, we aimed to explore students’ current SM utilisation rate and their attitudes and beliefs about the role of online teaching via SM tools as a substitute for regular classroom teaching. Further, many of the teachers and students have not been officially trained for online education; therefore, the role of SM tools in facilitating knowledge transfer remains known. Thus, the present study aims to (1) evaluate Indian physiotherapy students’ perceptions on the accessibility and acceptability of SM tools as a direct teaching pathway between faculties and students, and (2) assess the efficacy of SM tools as an educational medium in imparting knowledge. We hypothesize making online teaching a better implementable model and provide further insights to facilitate the transformation.

### Design

The data for the study was collected between June 2020 and September 2020. The study was divided into two parts. The first part was a cross-sectional, observational study and utilised an anonymous Google questionnaire (in English, Annexure 1). The items in the questionnaire were developed from previous questionnaires that have analysed the impact of COVID-19 lockdown on students’ academic performance [21] and usage of SM applications [17]. The questionnaire was developed to collect information regarding the demographic details of the participants (gender, age, place of residence, academic enrolment, and parents’ educational and financial status), as well as their prior experience in using online media for learning, perceptions on the role and impact of online teaching during the pandemic, and experiences and barriers while using SM tools. The questionnaire briefly stated the aim of collecting these data at the beginning. The final questionnaire for this study comprised 27 questions regarding the students’ SM utilisation frequency and views on online learning (four open-ended and remaining closed-ended), in addition to questions about the demographic characteristics of participants.

In the second part, we used an open label randomised comparative design to evaluate knowledge change through an online examination, which was administered 10 min before and after a short session delivered through two different SM platforms (namely, Google Meet and YouTube) and email.

### Participants

Full-time undergraduate physiotherapy students belonging to any geographical location within India were eligible to participate in the first phase of the study. There was no restriction in terms of participation in the study, except that students were asked to maintain sufficient battery and data backup for the online session. The questionnaire link was shared with participants through emails, WhatsApp, and other SM applications. Faculty members and colleges were requested to invite their students to participate in the survey through email or other SM tools. Online questionnaires regarding the use of SM tools were open between the months of June and July 2020.

For the second part of the study, the participants attended online classes via SM tools. The invitation to participate was distributed through emails to physiotherapy students of two institutions. The students who provided consent were randomly divided into three groups: Google Meet (group 1, 93 participants), YouTube (group 2, 93 participants), and email (group 3, 92 participants). These tools were chosen because they are currently the most popular SM platforms used by teachers.

### Intervention

A total of 278 participants responded to the invitation and consented to participate in the short online teaching programme on ‘Disability Models’ that was a part of their course curricula. This content was delivered (in English, 35 min) through the aforementioned SM platforms by a teacher with more than 22 years of classroom teaching experience. Participants in groups 1, 2, and 3 received the learning content via Google Meet (live teaching), YouTube (video), and as an email attachment (PDF), respectively. For sharing on YouTube, a video was recorded and not shared publicly until the day of evaluation. To maintain uniformity, all three modes of teaching were conducted simultaneously.

### Outcomes

Demographic data of the participants included gender, age, area of residence, financial status of their families, prior experience of SM use, and attitudes and beliefs of students regarding the use of SM tools in the online teaching context.

Knowledge transfer was measured using an online examination (10 questions) that was administered before and after the education programme. Questions were in a multiple-choice format, with one correct answer and four distractors for each question. The invitations to answer the pre-test and post-test questions via a Google Form were sent to all the participants by email 15 min before and after the delivery of course content. All the participants were instructed to submit the answers within 10 min; responses received after 10 min were not accepted. The same questions were used before and after the education sessions, except that the order of the questions along with their answers and distractors were changed to avoid answers based on pattern recognition. Each question was allocated 2 marks. No marking was made for not answering the question, and no negative marking was made for wrong responses.

### Data analysis

Basic demographics and ordinal responses to questions were recorded for all the participants. All data pertaining to knowledge transfer between the three groups were analysed using repeated measures ANOVA. There was one between factor (group) with two levels (groups 1, 2, and 3) and one within factor (time) with two levels (pre and post). The *P* value was set at 0.05. Data were analysed using Statistical Package for Social Sciences (SPSS 16.0 version).

## Results

A total of 627 participants completed the first part of the study. There were no partial/incomplete responses.

### Demographics

Both men (300, 47.8%) and women (327, 52.15%) participated in the first phase of the study. Majority of the respondents were < 30 years of age (89.6%). Parents of 51.0% of the respondents had education above graduate level. Families of 26.3% respondents had a monthly income of more than ₹25,000 (approximately US$ 340), families of 25.8% respondents had a monthly income of less than ₹15,000 (approximately US$ 203), and families of the remaining 47.9% respondents had a monthly income between ₹15,000 and ₹25,000. Of the total participants, 45.7%, 41.7%, and 12.4% had been living in the urban, rural, and semi-urban areas, respectively; 58.2% participants used to commute from their own home to college before lockdown, 14.4% participants used to stay in hostels, and 27.4% participants were living in rented houses. Moreover, 88.2% of the participants had moved back home during the lockdown, and 9.7% continued to stay in the rented/friends’ place.

Majority of the participants reported using smartphones to attend online classes (66%). Laptops, personal computers, and tablets were other electronic devices used for this purpose; 72% participants had prior experience of attending online classes/webinars, and 27.9% participants attended online classes for the first time during the lockdown. In addition, 51.7% participants reported being aware of contemporary tools/apps used for online classes; 46.7% participants spent < 4 h on surfing internet for learning during the lockdown, whereas 14.2% had never used the internet before lockdown for studies; this number decreased to 6.5% during the lockdown. Moreover, 28.4% participants reported that electronic devices used for attending online classes had developed some technical snags during the lockdown (which could not be repaired).

Most participants (74.6%) reported using SM for recreational purposes, and the most popular app was WhatsApp (94%), followed by Facebook (55.3%). On a scale of 1–5, with 1 being very good and 5 being very bad, 39.6% respondents rated 3 as the strength of internet connectivity; 14.2% of the respondents considered network connectivity to be very good (1), whereas 15.3% reported network connectivity to be very poor (5). Students reported spending from < ₹ 300 (approximately US$ 3.9) to ₹ 2000 (approximately US$ 27) per month towards data recharge, with more than a half of the participants (50.1%) using approximately 1.5 GB data per day for online classes.

### Attitudes and beliefs towards SM as an online teaching tool (Fig. 1)

The participants reported WhatsApp to be the most frequently used tool for online learning (62.8%), followed by Zoom (39.1%), and Google Meet (32.7%). Other apps used for online teaching were Skype, Telegram, Cisco WebEx, Microsoft Teams, and IMO. The percentage of the participants who reported that they could clarify their doubts in online classes ‘sometimes’ was 60.9%, whereas 9.4% reported that their doubts were never clarified; 45.9% and 47.7% participants reported that their faculties were able to clear their doubts ‘always’ and ‘sometimes’, respectively. Overall, 71.1% participants believed that online classes have helped them in their studies. The percentages of the participants who strongly agreed, strongly disagreed, and took a neutral stance to the statement ‘social networking platforms are helpful for teaching’ were 21.4%, 9.45%, and 39.1%, respectively. Furthermore, 22.6% and 10.2% participants strongly agreed and strongly disagreed to the statement that ‘social networking platforms will be used for teaching/learning in the future’, respectively; 34.1% participants held a neutral view about the same. The perceived barriers in using SM tools are presented in Fig. 2; 18.2% participants reported no changes in their ability to focus in online classes compared with traditional classroom teaching, whereas 53.1% participants reported considerable changes in attention. Moreover, 58.2% of the participants reported changes in discipline during the online classes, and 19.1% participants reported having no disciplinary changes. The perceived advantages of using SM tools for teaching purposes are presented in Fig. 3; 58.5% participants opined traditional teaching to be superior to online teaching; 44.25% participants reported having developed health issues such as soreness and pain in eyes, less sleep, fatigue, irritation in the ears, headaches, pain and stiffness in the neck and back, and feeling irritated by the end of the day.

**Fig. 1:**
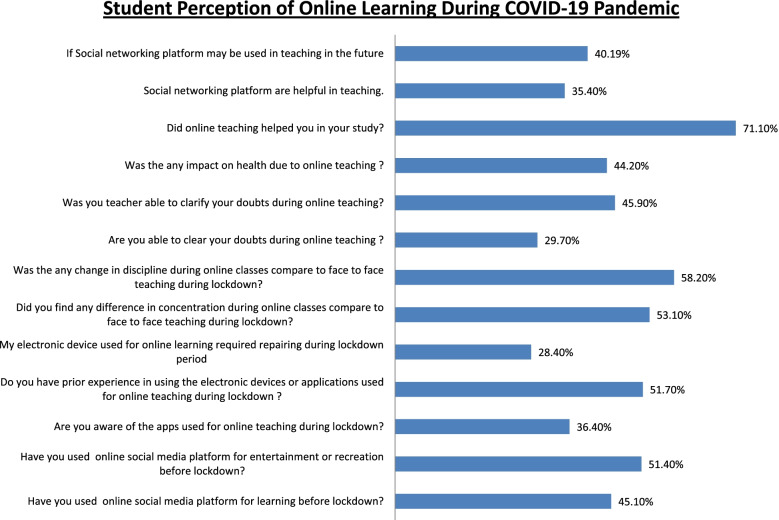
Student perception of online learning during COVID-19 pandemic

**Fig. 2:**
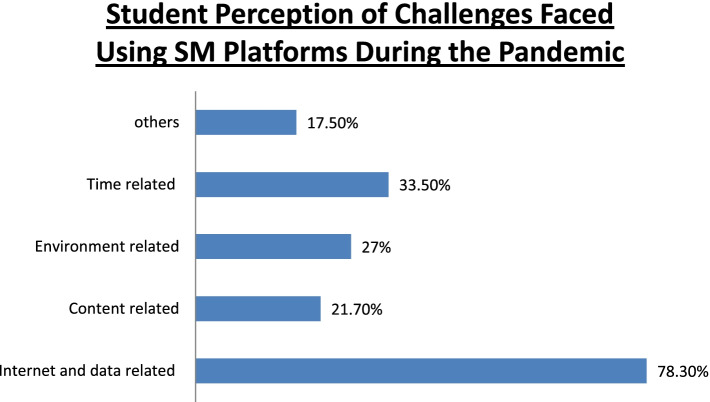
Student perception of challenges faced using SM platforms during the pandemic

**Fig. 3:**
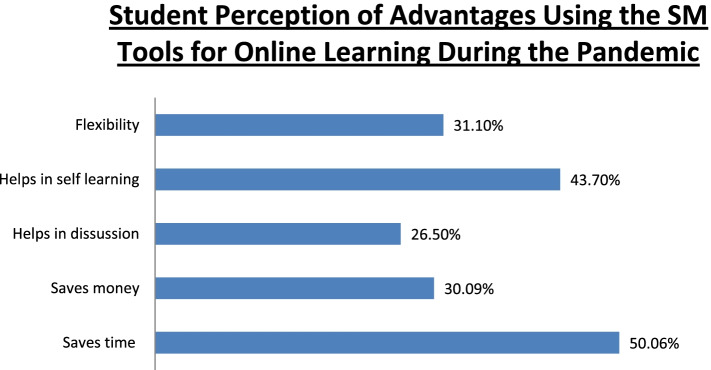
Student perception of advantages using the SM tools for online learning during the pandemic

### Knowledge transfer

Out of the 278 participants who enrolled for attending the course content, 224 participants (80.57% response rate) returned the answers for both pre- and post-questions. Responses from the participants to either pre- or post-questions were not considered. The mean pre-post scores are presented in Table 1. The results of ANOVA showed that the difference in knowledge transfer before and after the classes was nonsignificant (*p* > 0.05), both within and between the groups (both *p* > 0.05).

**Table 1:** Pre- and post-test scores: means, standard deviations, and confidence interval between groups

Group	Pre-score Mean (SD)	CI	Post-score Mean (SD)	CI
1 (68 responses)	10.76(3.32)	9.86–11.66	12.45 (4.42)	11.32–13.57
2 (82 responses)	9.67 (3.14)	8.77–10.57	12.37 (4.02)	11.32–13.42
3 (74 responses)	10.00(2.92)	9.13–10.86	12.19(4.16)	11.14–13.24

*SD* standard deviation, *CI* confidence interval

## Discussion

The results of the present study are important as students’ perceptions are vital to ensure effective e-learning and online learning, which may remain the norm even after the pandemic. The results indicate that though physiotherapy students hold positive attitudes towards the use of SM tools, the incorporation of SM into learning and teaching practices has to be carefully planned. The study demonstrates a high level of engagement by students with different SM tools.

The frequent use of SM tools by students is consistently reported across literature. For example, the use of SM increased from 12% in 2005 to 90% in 2018 [22]. Students reported using a wide usage of electronic devices such as tablets and smartphones. WhatsApp was reported to be the most popular app in this study; these conclusions are similar to the results of a study conducted in a Bahrain university [23]. However, WhatsApp has been criticised for its inability to find and utilise resources as the app is unique to the smartphone environment [24].

Few studies have explored the role of SM among undergraduate physiotherapy students in the UK. One study noted that students have developed confidence in using SM tools; SM tools such as Twitter can complement and modernise undergraduate physiotherapy education. The study also predicted that online education using SM tools will be a part of physiotherapists’ learning, and the physiotherapy students will use the technologies in their daily lives [25]. Another qualitative study reported that SM is an integral part of students’ daily lives and an adjunct to learning practices [26]. Considering the mixed evidence of SM tools, future online classes should be built with a well-designed course content and delivered by well-prepared instructors and using an appropriate technology [27].

The participants in the present study were from different geographical locations and economic backgrounds. Probably, the present study results have been influenced by the participants from rural areas, who could have faced technical problems related to electricity, internet connectivity, and inadequate computer labs/computers/laptops [28]. The strength of internet connectivity is a crucial factor for remote learning. According to a report, internet connectivity issues increased from 35% before the lockdown to 40% during the lockdown, with more than 82% of the surveyed people indicating that they could not fix their internet or connectivity issues. Good connectivity depends on the socioeconomic status and location. Rural households with no broadband connectivity face more connectivity issues. This disruption during online teaching can inhibit cohesive learning growth, which has led to a 43% reduction in the rate of students attending online classes and 44% reduction in the rate of students completing a lesson plan, resulting in an average 5-month setback [28, 29]. Problems with internet connectivity could result in students facing issues such as losing Internet connection and poor audio or video during an online class. These issues may be critical if the internet does not function in certain areas of the home, or if more than one person accesses from the same connection or because of reduced speed of connectivity during peak hours.

Students from economically weak backgrounds may not be able to afford online learning. A study reported that many countries face the problems of a stable internet connection and access to digital devices. Lack of parental guidance may be another issue when parents could not assist their children in classes [29]. According to a study, students face challenges such as socioemotional imbalance, difficulties adjusting to daily life activities at home, and financial burden [30] during the pandemic. A study from Bhutan reported that students have to assist their parents in farm during morning hours and look after their ailing parents/relatives [29]. Another study reported that students face difficulty in balancing their work, family, and social lives in an online learning environment [31].

The results of this study are similar to those of another study [32], which reported that students use 1.5–2.0 GB data per day and expiry of data is a major problem in continuing online classes. In another study, students belonging to weaker sections of the society have expressed their concern over the cost of data packages [29].

The results of our study indicated that knowledge transfer associated with the delivery of selected content was nonsignificant. Our results are in contrast to the conclusions of other studies that have reported the efficacy of online learning comparable to that of face-to-face learning. A study evaluated the efficacy of online and face-to-face teaching on the same content by the same instructor and instructional materials and observed no significant difference in the test scores and grades between the two groups [33]. Another study showed that students who engaged in a fully interactive multimedia-based e-learning environment outperformed and exhibited higher satisfaction than those who were taught in classrooms [34]. Another study reported that the passing rates of examinees increased after SM integration [35]. The results of a study [36] that analysed medical students’ perception of mandatory e-learning during the pandemic showed no statistical difference in the efficacy of face-to-face and online learning in increasing students' knowledge. However, e-learning has been considered less effective than face-to-face learning in improving clinical and social skills. Furthermore, the participants in this study felt less active during online classes. The results of the current study are in contrast to those of Shaheen et al. [18], who reported that SM tools can help enhance students’ academic performance. More than 9% of the participants reported that their doubts could not be clarified in the online mode. This might have reflected in the nonsignificant knowledge transfer results in examinations when there are problems in understanding the course content [37].

We hypothesise that the sudden shift to the online mode and the teachers' inability to design course content are the main reasons for the nonsignificant knowledge transfer results. The teachers are habituated to teaching in person [38], and a previous study showed that the teachers had to migrate to the SM platforms without receiving any training [39]. Different subjects require different approaches to online learning [40]. Students’ prior online experiences are not considered indicative of attending e-learning via online/SM classes. Moreover, classes via different SM apps were designed for 35 min, which is less than the stipulated 45-min duration recommended for digital teaching [38].

Mansell and Greene found that knowledge retention was affected in final year UK physiotherapy students as they do not actively engage with SM tools, despite using a variety of tools for learning; this finding is consistent with those of the present study. In addition, the authors concluded that educators should identify the most beneficial SM tools for learning [41]. The study was conducted during initial phases of the chaotic mandatory COVID-19 pandemic lockdown period. This period represents a time frame when only 30% of academic teachers reported having experience with online teaching [39]. During this period, most teachers had to change their teaching approach overnight [42]. A study conducted in Norway showed that though the students were satisfied with the make-shift online arrangement, receiving the education content online was a new experience for them, and they reported difficulties adjusting to the new teaching method [43].

The present study considered both synchronous and asynchronous e-learning strategies. The academics during the COVID-19 pandemic represent a period where the online teaching mode has become a necessity, which has further necessitated motivation and readiness change from its stakeholders. The conclusions of previous studies that SM tools were not developed for pedagogical purposes should also be considered [44]. Furthermore, it is unclear whether the pedagogical approach of classical instructional models that concentrate on individual processes of learning will be effective on SM platforms that concentrate on collaborative work and social interactions. Owing to the lack of effective interaction or collaboration during online classes, it may be best assumed that the ‘social features’ of ‘social’ media were not put in use. Therefore, the question whether ‘mandatory’ online learning for all subjects contributes to student achievement becomes critical. Online teaching through SM may be disadvantageous to students with less technical skills and those who can learn efficiently only through classroom learning [45]. Another factor for successful learning is to ensure successful communication between teachers and students. The class environment might provide teacher an opportunity to continuously monitor the students, which might not be available in teaching through online/SM tools as students may have to switch off their video for better connectivity. These tools may not provide a platform for addressing students’ problems, fear, or confusion. According to a study in China, the problems intrinsic to students such as lack of self-discipline contribute more to online learning outcomes compared with technical obstacles [46].

Detailed personal data could not be collected in this study; students’ circumstances during COVID-19 could have provided better information for arriving at a conclusion in this study. Moreover, the short- and long-term effects of campus closure on mental health of students and academic staff could not be ascertained. The stress associated with lockdown and the housing situations may also have contributed to the outcomes. Widespread closure of businesses and offices could have led to reduced income [29]. Additionally, because the students volunteered for the study, selection bias cannot be ruled out completely. The familiarity of resource persons with SM apps could be another limitation of this study. This, however, could depict the practical scenario in India existing at the time of COVID-19. The results of this study may not be generalised to other subjects and course contents. The subject familiarity of students to the course content even before the delivery of teaching cannot be ruled out. Furthermore, whether these results can be translated outside the pandemic period should be explored. Because we could not find other domestic studies that have analysed the role of SM tools in knowledge translation among physiotherapy/rehabilitation stream students, direct comparison of results was not possible. Some other limitations of the current work are: the internal consistency of questionnaire used was not computed; only students of two institutes were selected for the knowledge translation part of the study; and the study was concerned with ‘disability models’ only. Though the sample size was adequate for each group, anticipating a 10% drop-off in responses (incomplete/delay in responses), the drop-off was in the range 12–25%. Further research is recommended so that the effective role of SM in knowledge translation can be established. As perceptions may change over time, another study outside the pandemic is suggested for obtaining solid evidence. Future studies may consider identifying the appropriate contents that may be delivered via SM and evaluating the role of other factors such as distraction, disturbance in attention, changes in perception of time, and teachers’ fear of losing control over students in influencing study outcomes.

## Conclusion

The study demonstrated that a vast majority of students are engaged in SM and consider SM to be essential in online learning. However, multiple external and internal factors remain to be addressed before recommending SM as the possible means of improving the education scenario prevailing in India and elsewhere. Therefore, methods for improving the role of SM in online teaching should be explored. Training programmes for faculties for using SM should be considered. Online teaching via SM apps should be based on the principles of collaborative learning and sharing of knowledge resources. Furthermore, the appropriate course curricula that can be delivered through SM and the appropriate SM tool to achieve intended outcomes should be identified. Lastly, efforts should be made to create a conducive home environment for everyone, including students with special needs.

## Data Availability

The data that support the findings of this study are available from the corresponding author, [SG], upon reasonable request.

## Annexure 1

Link to the questionnaire used https://docs.google.com/forms/d/e/1FAIpQLSfwKfiFN8OpWpLHEvBEwwbwZbFvd-J1QmPNwpm1v0cmjWs10Q/viewform

## Abbreviations

SM: Social media
